# Retrospective evaluation of toceranib phosphate (Palladia®) use in the treatment of inoperable, metastatic, or recurrent canine pheochromocytomas: 5 dogs (2014–2017)

**DOI:** 10.1186/s12917-018-1597-7

**Published:** 2018-09-03

**Authors:** Margaret L. Musser, Kathryn L. Taikowski, Chad M. Johannes, Philip J. Bergman

**Affiliations:** 1 VCA Veterinary Referral and Emergency Center, Norwalk, CT USA; 2 Present address: Iowa State University College of Veterinary Medicine, Ames, IA USA; 3 Present address: Ohio State University College of Veterinary Medicine, Columbus, OH USA; 4 VCA Clinical Studies, Los Angeles, CA USA

**Keywords:** Pheochromocytoma, Toceranib, Canine, Metastasis, Recurrent

## Abstract

**Background:**

Effective treatment options for inoperable, metastatic, or recurrent canine pheochromocytomas are lacking. In humans, specific germline mutations exist that drive the development of pheochromocytomas. Pharmaceutical blockade of these abnormalities with small molecule inhibitors are an effective treatment strategy. Similar mutations may exist in the dog, and thus, treatment with similar small molecule inhibitors may provide a survival advantage. The purpose of this study was to assess the role of toceranib phosphate in the treatment of inoperable, metastatic, or recurrent canine pheochromocytomas.

**Results:**

Retrospectively, medical records of dogs that had a diagnosis or suspect diagnosis of a pheochromocytoma were reviewed for information regarding response to toceranib phosphate and overall outcome. Five dogs were identified that fit the inclusion criteria. All five experienced clinical benefit (1 partial response, 4 stable disease). Progression-free interval (PFI) for the dog with the partial response was 61 weeks. PFI for the two dogs with stable measurable disease were 36 weeks and 28 weeks. PFI in the two dogs with stable metastatic disease were at least 11 weeks and 18 weeks.

**Conclusions:**

Based on this limited series of dogs, the results suggest that toceranib may have biological activity in dogs with primary and metastatic pheochromocytomas. Larger studies are needed to define the use and response to toceranib in dogs with gross, microscopic, and metastatic pheochromocytoma.

## Background

Pheochromocytomas (PCs) are tumors comprised of neoplastic chromaffin cells of the adrenal medulla. Pheochromocytomas overproduce catecholamines, specifically epinephrine and norepinephrine, in an episodic manner, leading to intermittent clinical signs, including weakness, collapse, hypertension, panting, polyuria and polydipsia, and decreased appetite [1, 2]. Pheochromocytomas are uncommon in the dog, but must be considered malignant as severe consequences including invasion into the caudal vena cava, retroperitoneal hemorrhage, and metastatic spread can occur [2, 3].

Definitive treatment of a PC requires adrenalectomy [2]. However, anesthesia and surgical removal of an adrenal tumor is a high-risk procedure, carrying an overall 51% postoperative complication rate, which increases to 60% in dogs with PC [4]. These complications can include significant blood pressure variations, tachyarrhythmias, intraoperative hemorrhage, and intraoperative death [3, 4]. Vascular invasion into the caudal vena cava, reported in up to 82% of cases, [4, 5] complicates the surgical approach, but the impact on survival in these cases is unclear [4–6]. A median survival time of 53 weeks has been reported in dogs following surgical removal of a PC [7], with some living for 2–3 years [8, 9]. However, some PCs are not amenable to surgical removal or surgery is contraindicated due to the presence of widespread metastatic disease [6]. The benefit of medical treatment in these cases is unknown.

Pheochromocytomas affect 2 to 8 per million people per year [10, 11] and are biologically similar to those that affect dogs [1]. Standard of care is surgical removal, which often results in a normal lifespan for those individuals with local disease. However, 10–30% will develop distant metastatic disease, and in the 6.5 to 16.5% of patients that develop recurrence after surgical removal [12–14], 50% develop distant metastasis [14]. In the case of any distant metastasis, only 40–60% of patients survive an additional 5 years [1, 15]. These advanced-stage malignant PCs are resistant to chemotherapy and radiation therapy, and the side effects of treatment often outweigh the benefits [16].

In humans, hereditary germline mutations, and abnormally regulated angiogenesis and oxygen metabolism pathways, drive the pathogenesis of many metastatic PCs [17]. Therefore, recent PC research and clinical trials have focused on drugs that target these specific abnormalities. Sunitinib malate is a small molecule inhibitor that targets multiple tyrosine kinase receptors, including c-KIT, FLT3 and RET, and those that help to drive angiogenesis including vascular endothelial growth factor receptors (VEGFR) 1 and 2, and platelet-derived growth factor receptor-β (PDGFRβ) [12, 17, 18]. Recent research suggests that use of sunitinib in human patients with advanced PCs will result in tumor size reduction, disease stabilization, and improvement of hypertension [16, 17, 19, 20].

Toceranib phosphate is a tyrosine kinase inhibitor (TKI) available in veterinary medicine that is molecularly similar to sunitinib, and blocks the same key receptors associated with angiogenesis: VEGFR and PDGFR [21, 22]. Thus, it may be a good treatment option for canine patients suffering from advanced PCs that are biologically similar to human PCs, or in those patients where surgical removal is not a viable treatment option. This study aimed to determine if a response to toceranib is present in dogs with advanced inoperable, recurrent, or metastatic PCs. Based on the human literature, we hypothesized that dogs with a measurable PC or metastatic disease would have a biological response to treatment with toceranib.

## Methods

Medical records of dogs diagnosed with a PC and treated with toceranib were collected and reviewed via solicitation through the American College of Veterinary Internal Medicine Oncology Listserv, an e-mail based forum for oncology diplomates. Only dogs with complete records regarding signalment, history, initial complaint, physical examination findings, abdominal and thoracic imaging, treatment with toceranib for at least 10 weeks, and with adequate follow-up including records of side effects, were included in the study. The diagnosis of a PC was based on histopathology of the surgically removed tumor, or suspicion of a PC based on elevated plasma or urine metanephrine or normetanephrine levels as previously described [3, 23–27].

Data collected included breed, weight, clinical signs at diagnosis and duration of clinical signs, findings of thoracic and abdominal imaging, histopathology results, blood pressure, results of fundic examinations, urine metanephrine/normetanephrine levels if available, evidence of cardiac arrhythmias, therapy prior to the start of toceranib, reason for toceranib use (inoperable measurable disease, microscopic disease, treatment of recurrent disease, metastatic disease, or maintenance after completion of other therapy), toceranib dose and dose schedule, duration of toceranib treatment, best response to treatment using previously defined criteria [28], side effects induced by toceranib using the veterinary cooperative oncology group-defined criteria [29], concurrent chemotherapy and supportive medications, concurrent diseases, and patient outcome.

Progression-free interval (PFI) was calculated for all dogs and defined as the time from the day of first toceranib treatment to the time of disease progression (defined as either local regrowth, local progression, and/or metastasis), to the time of toceranib discontinuation due to adverse effects, or to the time of data submission. For dogs with measurable disease, as in previous publications describing the use of toceranib in dogs, clinical benefit (CB) was determined by best response to therapy and was defined as a complete response (CR) or partial response (PR) of any duration, or stable disease (SD) of at least ten weeks in duration [30].

Response to therapy was evaluated via serial abdominal ultrasounds, thoracic radiographs, and in one case urine catecholamine measurements. Animals experiencing SD less than 10 weeks in duration or progressive disease (PD) as their best response to toceranib were not classified as achieving CB.

## Results

Data for 6 dogs from 5 different sites were received. Five dogs were identified that fit the inclusion criteria (Table 1).

**Table 1:** Summary of patient characteristics and response to toceranib

Patient	Breed	Treatment of Measurable Disease or Metastatic Disease	Response	PFI (weeks)	Reason for Toceranib Discontinuation	Alive?
1	Shih Tzu	Measurable	SD	36	Diarrhea	Y
2	Aus. Terrier	Microscopic/Metastatic	SD	18	Ongoing	Y
3	Pomeranian	Measurable	SD	28	Ongoing	Y
4	Dachshund	Measurable	PR	61	Ongoing	Y
5	Pit Bull	Metastatic	SD	11	Lameness	N

PFI: Progression free interval

The sixth dog did not have adequate evidence of a PC diagnosis, and so was excluded from analysis. There were four spayed females and one castrated male. Breeds included one of each: Shih Tzu, Australian Terrier, Pomeranian, Dachshund, and Pit Bull. Mean weight was 12.1 kg (range 2.6–29 kg) with a mean age of 11.5 years (range 9.5–14 years). Clinical signs at diagnosis were varied, but included prolonged gastrointestinal signs (vomiting, diarrhea, decreased appetite, and weight loss) in three dogs, and exercise intolerance, heavy panting, and polyuria/polydipsia in two dogs. One dog was non-clinical and the PC was an incidental finding during work-up of a concurrent disease. Excluding this dog, clinical signs for the other four dogs were present for a mean of 22 weeks (range 1–52 weeks).

Physical examinations were unremarkable in all dogs. No cardiac arrhythmias were noted in any dogs at any time. A fundic examination was performed in four dogs, which was normal in all patients evaluated.

Abdominal radiographs were taken in one dog, which revealed mild hepatomegaly and a small liver mass. An abdominal ultrasound was completed in all dogs; all were diagnosed with a right-sided unilateral adrenal mass. Three of the dogs had evidence of caudal vena caval invasion. One additional dog developed a recurrent adrenal mass with invasion of the caudal vena cava one year after initial PC diagnosis and surgical treatment. Additional ultrasonographic findings included mesenteric lymphadnopathy concerning for metastatic disease (*n* = 1), an incidental splenic mass (*n* = 1), an incidental caudal abdominal mass (*n* = 1), mild hepatomegaly consistent with vacuolar hepatopathy (*n* = 3), renal cortical hyperechogenicity consistent with degenerative renal changes (*n* = 3), mild pancreatic enlargement and hyperechogenicity consistent with chronic pancreatitis (*n* = 1), scant peritoneal effusion (*n* = 1), mild urinary bladder wall thickening (*n* = 1), and one patient with multiple ventral caudal abdominal subcutaneous nodules suspected to be metastasis from a previously diagnosed mammary carcinoma (given location and the reported subcutaneous metastatic rate of 30% for canine mammary carcinomas [31]; diagnostic sampling was not pursued).

An abdominal CT scan was completed in two dogs for further evaluation of the adrenal mass. This confirmed the presence of the suspected adrenal mass in both dogs. In addition, in one patient invasion of the adrenal mass into the phrenicoabdominal vein and right liver was identified, which was not clear on the abdominal ultrasound completed prior to the CT scan.

Two dogs were diagnosed with adrenal PC based on elevated urine catecholamine levels evaluated through Marshfield Labs. One dog had an elevated normetanephrine/creatinine ratio (2268 μg/g creatinine; reference interval [RI] 28–380 μg/g creatinine). The other had an elevated norepinephrine/creatinine ratio (0.152 μg/mg creatinine; RI 0.001–0.037 μg/mg creatinine) and an elevated dopamine/creatinine ratio (0.1 μg/mg creatinine; RI 0.00–0.031 μg/mg creatinine). The remaining three dogs did not have urine or plasma catecholamine levels evaluated.

Full staging (thoracic and abdominal imaging) was completed for all dogs, which revealed that at diagnosis three dogs had evidence of suspect metastatic disease. One dog had pulmonary metastatic disease based on CT (thoracic radiographs were not completed). The remaining four dogs were free of pulmonary metastatic disease based on thoracic radiographs. One dog had evidence of several enlarged mesenteric lymph nodes on ultrasound concerning for early metastatic disease (as mentioned above). CT scan identified a suspect bony lesion in L1 of one additional dog which was concerning for metastatic disease. No dogs had evidence of multiple endocrine neoplasia syndromes type 1 or 2.

Three dogs underwent surgical removal of their adrenal tumors and had a histologic diagnosis of PC. Additional surgical findings included a caudal abdominal lipoma, a splenic hematoma, and chronic hepatopathy (abnormalities noted previously on ultrasound, as described above). One dog that was treated surgically had evidence of caudal vena cava invasion at surgery, but not during the planning abdominal ultrasound. A CT scan was not completed in this dog. The three dogs that received surgical treatment also had blood pressure readings taken prior to and after surgical removal of the pheochromocytoma. Mean blood pressure prior to surgery was 135 mmHg (range 72–204 mmHg). Mean blood pressure after surgery was 113 mmHg (range 80–151 mmHg).

In the three dogs that had surgical removal of their PC, toceranib therapy was started at the time of recurrent, inoperable disease in one dog, for adjuvant treatment of incomplete surgical margins and the development of pulmonary metastatic disease in the second, and for treatment of pulmonary metastatic disease in the third. In the remaining two dogs, toceranib was given to treat inoperable measurable disease.

All dogs received toceranib at a starting target dose of 2.75 mg/kg orally, rounded up to the nearest available tablet size, every other day (*n* = 1) or Monday, Wednesday, and Friday (*n* = 4). At the time of data submission, three dogs were still receiving toceranib; two had treatment discontinued due to adverse side effects (one due to grade 2–3 lameness; one due to grade 2 diarrhea, grade 2 anorexia, grade 2 lethargy, and grade 3 liver enzyme elevations). Two of the three still receiving treatment had been on continuous toceranib for seven and eight months. The third had been on toceranib for a total of 17 months, including a one-month treatment break following the development of an elevated urine protein/creatinine ratio, which resolved after the treatment break. The two patients no longer on toceranib had been treated for a total of six and nine months. One patient was treated concurrently with metronomic cyclophosphamide (dog 5). Additional supportive medications included enalapril (*n* = 3), metronidazole (*n* = 2), maropitant (*n* = 1), famotidine (*n* = 1), s-adenosylmethionine and silybin A + B (*n* = 1), amlodipine (*n* = 1), carprofen (*n* = 1), gabapentin (*n* = 1), tramadol (*n* = 1), and omeprazole (*n* = 1).

Four dogs had a normal blood pressure prior to starting toceranib (one dog was not evaluated). During toceranib treatment, two dogs developed hypertension (defined as a mean systolic blood pressure of > 160 mmHg as previously described [32]) greater than 220 mmHg. Enalapril was started in both dogs which resulted in the normalization of the blood pressure. In both dogs enalapril was continued for the duration of toceranib therapy.

Three dogs required a treatment break, discontinuation, or decreased dose due to adverse side effects. One dog developed grade 2 diarrhea, grade 2 anorexia, grade 2 lethargy, and grade 3 liver enzyme elevations that progressed while on toceranib. Several drug holidays were pursued, which resulted in temporary control of adverse effects, but even at a lower dose the gastrointestinal signs returned and toceranib was ultimately discontinued after nine months of treatment. One dog developed grade 1 diarrhea and grade 3 proteinuria. Enalapril was started for the proteinuria and a one-month break was given. This resolved the proteinuria and toceranib was restarted at a lower dose (1.9 mg/kg PO Monday, Wednesday, Friday). One dog experienced grade 2–3 lameness which resulted in toceranib discontinuation (Table 1).

Clinical benefit (CR, PR, SD) was noted for all five dogs (Table 1). Four of the dogs treated with toceranib had SD. For these four dogs, PFI was determined to be at least 11, 18, 28, and 36 weeks. In one of these dogs, response was evaluated via an additional urine normetanephrine/creatinine ratio which showed normalization of the ratio following the start of toceranib treatment (levels decreased from 2268 μg/g creatinine to 326 μg/g creatinine; RI 28–380 μg/g creatinine). The fifth dog had a PR with a PFI of 61 weeks that as of data submission is on-going.

At the time of data submission, four dogs were still alive. One dog (dog 5) had been humanely euthanized due to progressive metastatic pulmonary disease 23 months after surgery to remove the primary PC, and 11 months after starting toceranib therapy for metastatic disease. The dog was on a total of six months of toceranib which was discontinued five months prior to euthanasia due to lameness and negative impact on quality of life.

## Discussion

The aim of this retrospective study was to evaluate the biologic response of dogs with inoperable, metastatic, or recurrent PCs to standard toceranib treatment. Beyond surgical removal, which is the treatment of choice, information about adjuvant therapy is lacking. The best treatment practices for inoperable, recurrent, or metastatic tumors is also unknown. Based on this limited case series, it appears that toceranib may have biological activity in dogs with microscopic and macroscopic PCs (primary or metastatic disease), and that toceranib might be a reasonable treatment option for owners who are hesitant to pursue surgery, for tumors that are not amenable to surgical removal, and for dogs with distant metastatic disease.

Pheochromocytomas in humans and dogs are clinically similar. Optimal treatment centers on surgical removal, with limited treatment options for inoperable tumors, those that are metastatic, and those that are recurrent. In humans, intensive chemotherapy, radiation therapy, and radiopharmaceutical agents (^131^I-metaiodobenzylguanidine) result in palliation of symptoms, but do not have an impact on survival [16]. Tyrosine kinase inhibitors that target c-KIT, FLT3, RET, VEGFR 1 and 2, or PDGFRβ appear to have the best biological response, reported to be around 60% [16, 17, 19, 20].

In humans, hereditary germline mutations and mutations in the von Hippel-Lindau (VHL) gene lead to the development of multiple neuroendocrine tumors including PCs [17]. An abnormally functioning VHL gene allows the activation of hypoxia-inducible transcription factors (HIF) which in turn promotes cell growth, angiogenesis, cell survival, and activation of VEGF and PDGF [33]. Similarly, mutations in succinate dehydrogenase (SDH) subunits B and D lead to the activation of HIF and the development of aggressive PCs [1]. Blockade of these mutations with small molecule inhibitors such as sunitinib via the VEGFR cell signaling pathway have led to improved quality of life, tumor size reduction, and control of PC-induced clinical signs such as hypertension [17].

One study has evaluated canine PCs, and the related paraganglioma, for SDH mutations. In that study 50% of PCs (3/6) had germline mutations in SDH, indicating that the disease may have some genetic similarities to humans [34]. Canine VHL mutations have been evaluated in canine renal carcinoma [35], but not yet in PCs, to the authors’ knowledge. However, due to the evidence of SDH mutations and similar pathogenesis, it seems reasonable to conclude that human and canine PCs may be similar in their development, and thus treatment with similar TKIs blocking the VEGFR pathway may offer a therapeutic advantage for our canine patients.

Despite these similarities, the reported treatment of PCs in dogs has been largely limited to surgical removal. One case report describes the use of the radiopharmaceutical ^131^I-metaiodobenzylguanidine for an inoperable PC in a Yorkshire terrier [36]. ^131^I-metaiodobenzylguanidine is an alkyl-guanidine derivative with a molecular structure similar to norepinephrine. Selective uptake by a PC will occur after intravenous administration. The dog experienced stable disease for one and a half months after the first injection, but five months later developed progressive disease. A second injection was administered but the patient died three weeks later, likely due to compression-induced bowel ischemia [32]. To the authors’ knowledge, there are no reported studies of chemotherapy or toceranib for the treatment of canine PCs.

Toceranib phosphate is an oral TKI approved for the use in dogs with Patnaik grade II or III, recurrent, cutaneous mast cell tumors with or without regional lymph node involvement. However, it appears to have efficacy against multiple tumors types, including various neuroendocrine tumors [30]. It is known to block multiple receptors including VEGFR and PDGFR [21] that stimulate angiogenesis, a known driver of human PCs.

Three dogs developed grade 1 or 2 clinical toxicities, which is in keeping with the previously reported toxicity rate for toceranib of 77.6% [30]. In addition, the toxicities observed, such as diarrhea, decreased appetite, lameness/muscle weakness, proteinuria, and hypertension, were those previously associated with toceranib [30, 32]. Neutropenia was not reported in this group of dogs. Two dogs had effects that were severe enough to warrant discontinuation of toceranib, and an additional patient required a significant dose reduction due to grade 3 proteinuria. In a recent study evaluating the use of toceranib in solid tumors, 20% of patients required a drug holiday or dose reduction, although this reduction did not appear to affect efficacy [37]. The number of dogs in this study that required discontinuation of treatment was much higher, but that is likely due to low patient numbers and lack of statistical power.

Two dogs developed hypertension, one of which was the same dog that developed grade 3 proteinuria. Hypertension is a known side effect of toceranib and has previously been reported to occur in 37% of treated dogs [32]. Hypertension is also a well-known side effect in humans treated with sunitinib. In a systematic review of the side effects associated with TKIs for the treatment of gastrointestinal stromal tumors, which inherently should not cause hypertension like PCs, 36% of human patients developed hypertension while on sunitinib [38]. In addition, in a study of humans with PC and paragangliomas treated with sunitinib, 82% had hypertension at presentation, and 35% had exacerbation of their hypertension while on sunitinib [17]. The underlying pathogenesis of TKI-induced hypertension is unknown. Prevailing theories include: (1) VEGF blockade, which prevents nitric oxide synthetase production of nitric oxide, allowing endothelin driven vasoconstriction; (2) decreased VEGF which may remodel capillaries and lead to endothelial dysfunction; or perhaps (3) TKIs may induce tumor cell apoptosis, leading to release of stored catecholamines and thus hypertension [39]. Recently, it has been determined that the development of hypertension while on a TKI is a biomarker of efficacy in some human patients treated with sunitinib [40]. In the current study, the two dogs that developed hypertension after starting treatment with toceranib both had a prolonged PFI (28 weeks and 61 weeks, respectively), and include the one dog that had a PR. One could make the argument that the development of hypertension may infer a more robust and durable response to toceranib.

There are many limitations to the current study due to its retrospective nature and small patient numbers. Histopathologic confirmation of a PC diagnosis was not obtained in all dogs. However, this is often the case in practice as a presurgical presumptive diagnosis of PC is frequently based on clinical signs and abdominal imaging alone [1, 2, 8]. The addition of urine normetanephrine/creatinine ratio measurement may aid in the diagnosis of pheochromocytoma, allowing for appropriate targeted therapy. However, sensitive and specific reference ranges have not been established to confirm the diagnosis of a PC in all clinical situations [23–27]. In addition, this diagnostic is not readily available at most laboratories (available, to the authors’ knowledge, through Marshfield Labs, ARUP Labs, and some local human hospitals). Similarly, metastatic disease, although suspected in several dogs, was not confirmed at the time of development or on necropsy. In addition, the natural course of this cancer in these dogs prior to diagnosis and the impact of toceranib on that natural progression, is unknown.

One dog received metronomic cyclophosphamide in addition to the toceranib. Although cyclophosphamide is used to treat humans with PC in a combination protocol with vincristine and dacarbazine, use as a single agent has not been reported [41]. In addition, low-dose metronomic cyclophosphamide (used in the dog reported herein), has not been evaluated for efficacy in human or canine PC. The impact of the addition of cyclophosphamide on this dog’s outcome is unknown, but may have positively contributed to the stable disease observed (dog 5). Therefore, given these caveats, it is impossible to definitively conclude that toceranib has clinical benefit for canine PCs.

## Conclusions

Despite the aforementioned limitations, this study suggests that toceranib may offer clinical benefit to dogs with PCs. Additional studies investigating the presence of germline mutations similar to those identified in humans could help to support this conclusion. Furthermore, prospective studies are needed to better elucidate the potential role of toceranib in the treatment of both macroscopic and microscopic PCs.

## Abbreviations

CB: Clinical benefit
CR: Complete response
HIF: Hypoxia-inducible transcription factors
PCs: Pheochromocytomas
PD: Progressive disease
PDGFRβ: Platelet-derived growth factor receptor-β
PFI: Progression-free interval
PR: Partial response
RI: Reference interval
SD: Stable disease
SDH: Succinate dehydrogenase
TKI: Tyrosine kinase inhibitor
VEGFRs: Vascular endothelial growth factor receptors
VHL: von Hippel-Lindau gene
